# The Promising Impact of a Virtual Music Intervention for Persons With Dementia

**DOI:** 10.1093/geroni/igab046.497

**Published:** 2021-12-17

**Authors:** Cynthia McDowell, Jannell Walton, Debra Sheets, Andre Smith, Robert Stawski, Stuart MacDonald

**Affiliations:** 1 University of Victoria, Port Moody, British Columbia, Canada; 2 University of Victoria, Victoria, British Columbia, Canada; 3 Oregon State University, Corvallis, Oregon, United States

## Abstract

This study examines the within-person association between negative affect and global cognitive function for persons with dementia. Participants (n=33) engaged weekly in the Voices in Motion (ViM) sociocognitive choral intervention spanning up to 18-months and 9 individual assessments. Results revealed a significant time-varying association whereby within-person improvements in negative affect dynamically covaried with improvements on the Mini Mental State Examination (p<.05) across months of participation. These findings imply that, within-persons, reducing comorbidities associated with dementia (e.g., elevated negative affect) through participation in a lifestyle intervention, may facilitate increases in cognitive function. During the current pandemic, ViM transitioned to an online choir allowing for individuals to continue participating in the intervention and to maintain necessary social connections. The discussion focuses on the implications of this virtual choral intervention and the importance of modifiable risk factors such as negative affect and social isolation on the maintenance of cognitive health.

